# A Case Analysis of INFOMED: The Cuban National Health Care Telecommunications Network and Portal

**DOI:** 10.2196/jmir.8.1.e1

**Published:** 2006-01-27

**Authors:** Ann C Séror

**Affiliations:** ^1^Department of ManagementFaculté des Sciences de l’AdministrationUniversité LavalPavillon Palasis-Prince, Cité UniversitaireQuébec, QC G1K 7P4Canada

**Keywords:** Case analysis, Cuban national health care system, INFOMED, ideology, virtual infrastructure, health care markets, globalization

## Abstract

**Background:**

The Internet and telecommunications technologies contribute to national health care system infrastructures and extend global health care services markets. The Cuban national health care system offers a model to show how a national information portal can contribute to system integration, including research, education, and service delivery as well as international trade in products and services.

**Objective:**

The objectives of this paper are (1) to present the context of the Cuban national health care system since the revolution in 1959, (2) to identify virtual institutional infrastructures of the system associated with the Cuban National Health Care Telecommunications Network and Portal (INFOMED), and (3) to show how they contribute to Cuban trade in international health care service markets.

**Methods:**

Qualitative case research methods were used to identify the integrated virtual infrastructure of INFOMED and to show how it reflects socialist ideology. Virtual institutional infrastructures include electronic medical and information services and the structure of national networks linking such services.

**Results:**

Analysis of INFOMED infrastructures shows integration of health care information, research, and education as well as the interface between Cuban national information networks and the global Internet. System control mechanisms include horizontal integration and coordination through virtual institutions linked through INFOMED, and vertical control through the Ministry of Public Health and the government hierarchy. Telecommunications technology serves as a foundation for a dual market structure differentiating domestic services from international trade.

**Conclusions:**

INFOMED is a model of interest for integrating health care information, research, education, and services. The virtual infrastructures linked through INFOMED support the diffusion of Cuban health care products and services in global markets. Transferability of this model is contingent upon ideology and interpretation of values such as individual intellectual property and confidentiality of individual health information. Future research should focus on examination of these issues and their consequences for global markets in health care.

## Introduction

National health care systems are motivated by highly diverse ideologies giving rise to consumer-driven as well as social medicine models delivering widely varying quality of health care. Health care is defined here as the preservation of mental and physical health by prevention or treatment of illness through services offered by the health professions [1]. A health care system is a dynamic set of interconnected individuals, institutions, organizations, and projects offering products and services in health care markets [2]. The boundaries of such systems are increasingly difficult to identify. While many analyses, such as the annual reports of the World Health Organization (WHO), refer to national health care systems [3], diverse system boundaries may also be defined by overlapping corporate, professional, or other social entities.

Virtual infrastructures refer to (1) an environment characterized by overlapping distribution networks, systems brokerage functions, and the adoption of a software perspective emphasizing the devices and channels through which information is processed and distributed, as well as (2) a layer of abstraction between the computing, storage, and networking hardware and the software technologies that allow multiple operating systems to run on the same processor. This layer of abstraction leads to standardization and the support of legacy operating systems and applications on current hardware and software platforms. These infrastructures in turn are accessible through Internet websites and gateways designed to facilitate integrated use of the resources offered through virtual infrastructures. The adjective “virtual” thus describes any Web-based product, service, organization, or institution arising from the technical infrastructure defined above [4].

International trade in health care services and the globalization of national economies raise questions with regard to the emergence of institutional infrastructures in light of the deepening divide between the wealthiest industrialized nations and the developing world. Internet and telecommunications technologies are contributing to emergence of health care markets around the world. Research on economics and health care services has shown that national health care system performance is not directly related to gross national product (GNP) but rather is a function of variables describing rate of investment in public health as well as mechanisms for the equitable distribution of wealth [5-7]. Health care systems deliver widely differing services in terms of overall performance and per capita cost [3]. Thus, institutional and organizational configurations based on diverse ideologies are hypothesized to account for some of this variance. Ideology is the body of integrated assertions, theories, and aims that constitute a coherent sociopolitical system. Health care system ideology is expressed as the extent or manner of government and other stakeholders’ involvement in the financing, administration, and regulation of health care [8,9]. Indicators of such involvement include investment in health care, oversight and control of health care services, as well as ownership and governance of the health care system.

Research on the association between ideology and health care system structures, processes, and performance remains inconclusive [10-16]. Recent qualitative case studies conducted in China [17], South Africa [18], and countries in transition from communism [19] present mixed results. Health standards in China have improved under communist rule, while the transition from communism in Russia and Eastern Europe has resulted in short-term regression in such standards with some signs of improvements to come in the future. The South African democracy has seen significant regression in health indicators due to factors related to the AIDS pandemic as well as the high rate of violence in the country. The conclusions of these studies suggest that a more detailed analysis of the configurations of health care systems is required to understand dimensions affecting their internal coherence.

Consistent with Sen [5], Franco et al [11] found that democracy was more strongly associated with life expectancy, infant mortality, and maternal mortality than was GNP. Sen has pointed out that national health care system effectiveness measured on public health indicators such as life expectancy is not directly correlated with GNP, but rather that this relation is mediated through variables related to public investment in health care as well as mechanisms for the redistribution of wealth. This economic analysis is validated in the WHO’s rankings [3] of the general effectiveness of national health care systems.

For example, in 2000, the general performance of the US health care system was ranked 37th by the WHO and the Cuban system was ranked 39th of 191 member countries [3], while total health care expenditure per capita was estimated (at 2002 international dollar rates) at $5274 and $236, respectively [20]. The level of expenditure in the United States, highest among member countries of the Organization for Economic Cooperation and Development (OECD), is not reflected in the health care system performance measured as life expectancy among OECD nations [21]. Despite resource constraints [22], the Cuban national health care system has achieved a significant level of health care quality and equity as measured by population health criteria—the highest life expectancy in the Caribbean region as well as the highest concentration of physicians in the world. According to the United Nations Development Program, there were estimated to be 591 physicians per 100000 population in 2004 [23].

The inconclusive evidence for the association between health care system effectiveness and ideology may be the result of the wide variety of descriptive as well as quantitative methods used in the studies and the lack of a systematic approach to meta-analysis. However, it is also important to consider the complexity of health care systems and the proposition that system performance may be better explained by the internal coherence of the system, including the fit between system configurations and market ideologies as well as their integration in global networks and their adaptability to rapidly changing political and economic environments.

### Research Problem and Objectives

The Cuban strategy for an information society recognizes the critical importance of linkages among research activities and all economic sectors of activity, including health care. The accomplishment of this objective depends upon universal application of information technologies and development of national innovation systems and networks [24]. Extensive research has focused on the critical importance of proximate organization networks for knowledge creation and learning, particularly in health care and biotechnology [25-28]. This research has shown how diverse government, educational, research, and service entities contribute to effective research and development and service delivery. However, little research has examined specific institutional network configurations serving health care systems with the emerging roles of virtual infrastructures.

The Cuban system’s perspective on health care integrates evidence-based practice as well as medical and social science criteria for evaluation of system performance [29,30]. Of pivotal concern in analysis of the Cuban case are the socialist ideological principles upon which technological and institutional infrastructures are founded [9,31,32]. These principles, particularly social welfare priorities of free and equal access to health care and education for all Cuban citizens, affect the social and ideological pattern of interaction between telecommunications technologies and institutional choices within the society. Social behavior and community participation shape technological development by a process reinforcing institutional structures as well as organizational adaptation [33,34]. In the centralized Cuban social medicine model, unique social control structures suggest that the role of technology is significantly different from that in the free health care market driven by consumer demand [35].

Information and telecommunications technologies are changing the configuration and modifying the definition of sustainable health care system performance. Pressures for collaboration, data sharing, and access to distributed resources increase the focus on the interconnection of services both within and across institutions. Thus, both technological trends and commercial pressures foster service decomposition and distribution through networks rather than host-centric systems [2]. The pattern of medical informatics system evolution can be traced across three generations from system creation at the enterprise or institutional level beginning in the 1960s, through integration of enterprise architectures in the 1980s, to horizontal linkage and coordination across contemporary system boundaries. Effective contemporary medical informatics systems encompass components of all three generations [36].

The Internet and telecommunications infrastructures contribute to control mechanisms of health care management systems [37] through electronic markets, hierarchies [38], and heterarchies [39-41]. Electronic hierarchies are electronic linkages controlled by a centralized managerial system, as in the model of social medicine, while electronic markets supported by Internet and telecommunications networks foster competition among multiple buyers and sellers [42,43]. Heterarchies are complex systems of diverse interdependent entities. Institutional networks contribute to market dynamics based on the creation and supply of products and services (push) [42]. The performance of health care markets is founded substantially on the linkages among research and service delivery institutions as well as business enterprises.

While the Cuban national health care system and INFOMED form a rich context for the study of an integrated socialist health care environment, little previous research has focused on the unique characteristics of these social structures.

The objectives of this paper are to present the context of the Cuban national health care system since the revolution in 1959, to identify the configuration of INFOMED and its virtual institutional infrastructures using a qualitative research methodology, and to describe how these infrastructures contribute to international health care services markets. Ideological factors affecting the transferability of the Cuban model are considered.

## Methods

Qualitative case analysis is a research methodology particularly appropriate to the study of the health care sector [44]. Technological innovation and economic globalization drive rapid changes rendering nomological model identification more elusive. Idiographic case research methods are thus useful for rich descriptive analysis and assessment of complex health care management systems within their social, economic, and cultural contexts [43-46]. Multiple sources of data were used in this study, including published studies and research reports well as the Internet sites of the health care institutions under study and their network configurations.

The holistic level of analysis includes the health care system and its virtual environment. Chronological ordering of information shows how telecommunications and Internet strategies unfolded in the socialist ideological context of the Cuban national health care system.Network structure is defined as a system or configuration of relations among traditional and virtual institutions on the Internet. Properties of these information structures include attributes of institutions as well as the nature of relations among them, such as hierarchy and centralization [47,48]. Network configurations arising from these properties reflect social and institutional patterns of information management and control [43]. Qualitative analysis identifies linkages among traditional and virtual institutions and serves as a basis for mapping their configuration. Particular attention is focused on institutional configurations and e-commerce in global health care service markets.

## Results

### Historical Context

Since the Cuban revolution in 1959, Fidel Castro and the country’s leadership have pursued strategies to integrate national research and innovation policies through development of traditional institutions and, since 1990, virtual infrastructures [36,49-52].

- Development of a science base and infrastructure (1959-73): early transformation of the health care system and creation of integrated polyclinics (1963) to serve the Cuban population
- Elaboration of a centralized management model (1974-89): integration of information from various sources through institutional information architectures; introduction of community medicine (1974) and, subsequently (1984), the family doctor–and–nurse model
- Horizontal coordination and globalization through virtual infrastructures (1990-present): continued development of the Cuban social medicine model with emphasis on national infrastructure for institutional linkage of diverse sources of information and integration in international telecommunications infrastructures

In 1963, municipal polyclinics were first created to form the basic units of the Cuban health care system and to manage all health care activities within their jurisdictions, including workplaces, schools, and childcare centers. These activities were the first programs of the current community-based health care model. In 1965, both the National Center for Scientific Research (Centro Nacional de Investigaciones Cientificas) and the National Information Center for the Medical Sciences (Centro Nacional de Información de Ciencias Médicas de la República de Cuba, CNICM) were founded to serve the institutional needs of science with a priority on research in health care. The CNICM offered services for document distribution in Havana and throughout the country, as well as a system designed for the collection and analysis of information for health care evaluation. Coordination of these services contributed to universal access for all citizens as community-based social organizations encouraged participation in health-related activities such as vaccination, blood donation, and neighborhood clean-up efforts [53].

An evaluation of the municipal polyclinic model implemented in 1964 showed a lack of integration of health care activities across disciplines, persistence of curative over preventive priorities, lack of teaching and research opportunities in primary care, and inadequate coordination of polyclinic relations with hospitals and emergency rooms. Evaluation of this model led to the development of a new community medicine model. The system focus shifted at this phase from expressed morbidity to the preventive diagnosis of unexpressed morbidity by continuous assessment of risk factors associated with certain conditions, such as diabetes [53]. Continuous individual medical assessment and risk evaluation (dispensarización) transformed the earlier health care model and the activities of integrated municipal polyclinics [54]. In the period from 1971 to 1975, services for statistical analyses were integrated in the CNICM network [55].

Professors and medical residents increased their collaboration in polyclinic activities thus promoting opportunities for teaching and research in primary care. To further develop the focus on preventive medicine, a new holistic approach encompassing evaluation of social factors and preventive health care strategies was initiated in 1984 and later implemented throughout the country based on the *family doctor–and–nurse model* of medical practice. By 1984, CNICM had assumed the role of Cuban national coordinator for the Brazil-based Latin American and Caribbean Center for Information Sciences (BIREME), and preparations began to automate medical information services [55].

The information requirements of the Cuban national health care system continued to increase in complexity with the emergence of institutional networks and continuing emphasis on education and research. All of these factors contributed to further development of telecommunications infrastructures to support health care information, communication, and service delivery. These infrastructures reduced institutional health care costs in difficult economic conditions, including the collapse of the Soviet Union after 1989 as well as sanctions imposed by the US government [52,56,57]. INFOMED, the Cuban National Health Care Telecommunications Network and Information Portal (Red Telemática y Portal de Salud de Cuba), as well as academic telecommunications networks linking universities and research institutes became particularly critical to health care workers’ access to information. International organizations collaborated for the development of this network starting in 1992 when INFOMED was founded with the creation of the national network node in Havana. The United Nations Development Program, the WHO, the Pan-American Health Organization, and UNICEF made significant financial contributions to this effort [58]. The INFOMED network, later extended throughout the 14 Cuban provinces, made electronic access to important databases possible, including the US National Library of Medicine, the Cuban National Library of Medicine, and the growing collection of specialized Cuban medical journals such as ACIMED, the first Spanish language journal of medical informatics, founded in 1993 [55].

INFOMED developed collaborative projects with BIREME and offered training and assistance to other countries of the Caribbean and Latin American regions, such as Ecuador, Mexico, and Venezuela, where the Cuban health care model offers a reference for sustainable system development. The Virtual University project was inaugurated by the Ministry of Public Health in 1999 to improve continuing post-graduate medical training for more than 100000 Cuban health care professionals and to create an international center for post-graduate education in medicine and related disciplines [59]. These developments in the health care information system have contributed to the extension of the family doctor–and–nurse model of primary care, increased interdisciplinary integration of the activities of diverse health care actors, and emphasized continuous data collection, analysis, and dissemination throughout the system [60]. In 2002, INFOMED was awarded the Stockholm Challenge Prize in the health category for life-improving information technologies [61].

### INFOMED and the Cuban National Health Care System

The current Cuban model integrates the family doctor–and–nurse model and a community-based health care strategy while emphasizing the social relationships among patients, families, and physicians specialized in comprehensive general medicine. More than 30000 family doctors, each usually assisted by a nurse, serve neighborhoods of approximately 150 families whom they know intimately [62]. Community and family participation throughout the system, as well as continuous individual medical assessment (dispensarización), link the collective and individual levels of health care [54]. While population-level data are analyzed for performance evaluation and policy making, individual patient histories are maintained in paper files and archives. A project has been formulated to create passive electronic archives of patient histories more than two years old on CD-ROM disks. Paper files are considered critical for the legal record of individual patient care [63,64]. Qualitative and quantitative data are required for interdisciplinary medical practice, administrative coordination, community participation, and health care system evaluation. A key characteristic of the model is participation of the family as a social unit with attention to social morbidity as well as family culture and environment [53,65].

The integrated INFOMED network and the Cuban Ministry of Public Health (Ministerio de Salud Pública, MINSAP) assure both horizontal coordination and hierarchical control of the Cuban national health care system [56,57]. The hierarchical organization of MINSAP is comprised of 22 functional areas, including health statistics, hospitals, and ambulatory care, managed through the ministry and its board of directors as well as institutions at the national, provincial, and municipal community levels. At the municipal level, the people’s assembly, basic work groups (grupos basicos de trabajo), and the family doctor contribute to local health care management [66]. Government, health care institutions, and mass organizations such as youth and women’s groups are integrated in a distinctive social control system [35,67].

While MINSAP is largely responsible for hierarchical control, INFOMED is the vehicle for horizontal communication and coordination throughout the health care system. INFOMED also supports international collaboration and dissemination of information as well as the growing international trade in Cuban health care services. Specialized networks connect provincial information centers, research institutes, hospitals, and institutions of higher education. The virtual infrastructure maintained through INFOMED includes the Virtual Library (Biblioteca Virtual en Salud, BVS) and Virtual University (Universidad Virtual), the Health Information Observatory (Vigilancia en Salud), and key ministerial structures accessible through the portal as shown in the Figure.

The current mission of INFOMED is to develop an integrated telecommunications network for access and management of information and knowledge for improvement of clinical care, training, research, and health care management systems. Its mission is to improve the efficiency of the Cuban national health care system through development of an advanced electronic information infrastructure in order to foster communication and interaction between the international scientific community and Cuban health care workers, including clinicians, educators, administrators, professionals, and technicians [58]. Furthermore, INFOMED is designed to offer a virtual workspace and timely information access required for optimal performance without regard for physical location or the technical characteristics of work stations. Strategic objectives of the network include [58,68,69] the following:

- To facilitate electronic information access through the Virtual Health Library linked to provincial resources as well as regional and international databases available on the Internet
- To create an infrastructure of technical, organizational, and human resources for sustainable growth of INFOMED
- To facilitate continuing education for health care professionals through the Virtual University
- To maintain a continuous health information observatory through the National Office for Analysis of Health Care Trends
- To develop specialized telemedicine networks for services consistent with levels of telecommunications infrastructure throughout the country
- To support communication and create a virtual workspace linking health care institutions within Cuba and outside the country
- To develop software and implementation methods for projects designed according to the INFOMED model
- To promote Cuban scientific research and publication in the field of health information science

Technical personnel at both the national and provincial network nodes are specialized in network management, the Linux operating system [70], and system security. INFOMED experts create information products and services for the national health care system and assist regional information centers in the introduction of new information technologies. The telecommunications infrastructure of INFOMED consists of a national TCP/IP network for data transmission serving all entities of the national health care system. A public data transmission network links the national node and provincial nodes. INFOMED is connected to INFOCOM [71], the data transmission network of ETECSA, and CITMATEL [72], the Cuban Internet provider. INFOMED also possesses a national infrastructure connecting the medical science faculties of the 14 Cuban provinces for electronic messaging and access to electronic products and services. A telecommunications laboratory serves as a center to develop specialized expertise on computation, networks, website design, and information technology.

The Virtual Library integrates access to Cuban electronic publications in medicine and public health as well as important US, Latin American, and international publication initiatives. Medline and the US National Library of Medicine offer subscribed English language bibliographic databases while SCIELO, the Latin American Scientific Electronic Library Online, initiated in Brazil, offers medical journals by country of publication (Brazil, Chile, Cuba, Costa Rica, Spain, and Venezuela) in English, Spanish, and Portuguese.The INFOMED website offers a search tool, the reference locator for local, national, and international health information resources (Localizador de Recursos de Información de Salud) [73]. INFOMED also provides access to the Health Internetwork (HINARI), launched in September 2000 by the United Nations and the WHO to promote free institutional electronic access to medical publications in the developing world [74]. Thus, the Virtual Library integrates resources from the developed and developing world including the most advanced scientific research, accounts of medical experience in developing countries, and documentation of natural and traditional approaches to medicine.

The Virtual University is now part of the National Center for Medical Training through INFOMED and integrates all of the institutions of the Cuban national health care system, thus extending its institutional scope throughout the country [75]. This institution links the Cuban health care information and publication infrastructure with Cuban institutions for higher education, and it offers access to Cuban as well as international content such as the supercourse entitled Epidemiology, the Internet, and Global Health [76]. As part of the Virtual University, a Virtual Clinic offers expert consultation among the physicians and health care professionals associated with the University [77]. When authorized, consultations of particular pedagogical value are published for the benefit of other users of the clinic. The interactive design of the Virtual University promotes an information market for shared expertise and learning serving the Cuban national health care system as well as external markets [74,75].

INFOMED also serves evidence-based practice of medical specialties. An example is the Cuban Pediatric Surgery National Network (Red Nacional de Cirugía Pediátrica). The Cuban Ministry of Public Health designated the lead network institution, the Pediatric Teaching Hospital Octavio de la Concepción de la Pedraja of Holguín, in 2001 [78]. The objectives of the network are to develop and support the specialty of pediatric surgery throughout the country, to make available high-quality research results for the practice of this specialty, and to link the resources of all the participating health care institutions across the country. Local area networks of hospitals are linked to integrated services in radiology, endocrinology, and neurophysiology through the INFOMED portal. Regional experts in pediatric surgery have been identified for participation in discussion lists. These experts may participate in treatment of cases through collaboration with designated specialists or through collective consultation and analysis. The network also offers linkages to international discussion lists on topics relevant to pediatric surgery.

When appropriate, the network makes possible expert intervention in real-time treatment. Evidence-based protocols for best practice are developed using virtual analysis as well as face-to-face discussion for approval. Key features of the system include its intensive use of human resources, software development, and website design [79], a specialized virtual library, and alliances with provincial universities and enterprises. This model is under evaluation to be extended to other medical specialties.

**Figure 1:**
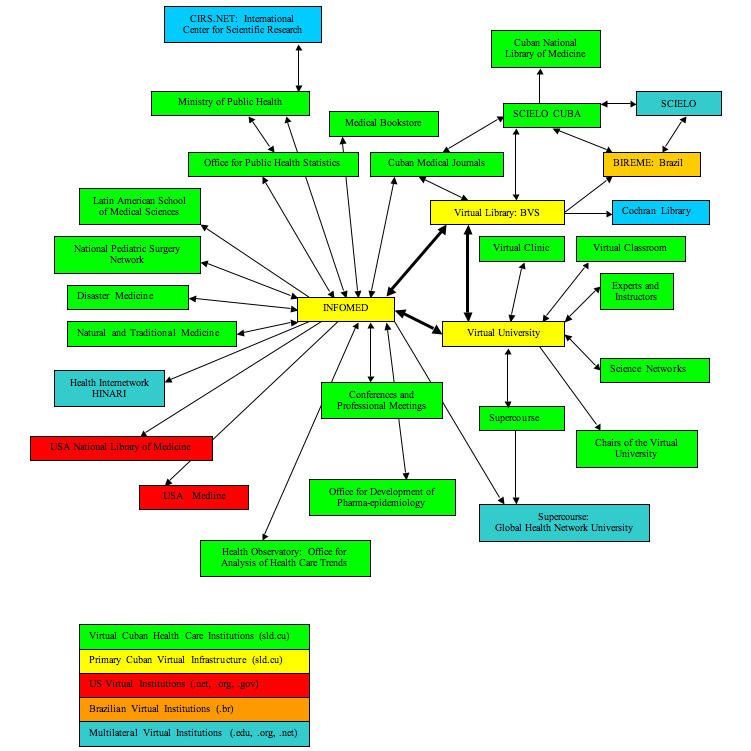
Institutional configuration of INFOMED and the virtual infrastructure of the Cuban national health care system

The Figure summarizes the configuration of INFOMED and the institutions of the Cuban national health care system. As discussed above, the primary virtual infrastructure includes INFOMED, the Virtual Library, and the Virtual University. Traditional political institutions such as provincial and municipal people’s assemblies ensure hierarchical control linked to MINSAP. The traditional and virtual infrastructures suggest the isomorphism of state governance with health care administration and a high degree of human resource intensity as evidenced by the critical role of the family doctor. Hierarchical control is maintained through the human resources of traditional institutions while horizontal coordination and institutional integration is accomplished through the virtual infrastructure of INFOMED. This system continues to evolve as the important parallel dynamics of institutional decentralization and network integration converge [69].

### Cuban International Trade in Health Care Services

The INFOMED infrastructure plays a key role in development of the Cuban contribution to international trade in health care services. Globalization of the health care sector is based on the decline in public sector expenditure, growing private health care enterprise, deregulation in insurance and telecommunications sectors, growing mobility of both consumers and health care service providers, and cross-border e-commerce for delivery of both health care products and services. There is also a high degree of variability in health care available across national health care systems. This variability affects consumer mobility as well as global patterns of international investment. The General Agreement on Trade in Services [80,81] suggests four modes of international trade in health services:

1. *Cross-border delivery* includes physical mail shipments of products or services such as lab analyses, pharmaceuticals, or clinical consultations as well as delivery through e-commerce channels or email.
2. *Consumption abroad* includes cross-border consumer mobility to obtain health care products or services.
3. *Commercial presence* refers to the establishment of health care entities and enterprises through foreign direct investment as well as diversification of international enterprises to extend commercial presence to other countries.
4. *Movement of health personnel* includes mobility of doctors, nurses, consultants, and administrative personnel to offer services across borders.

The Cuban national health care system contributes to health services trade in each of the four modes identified by the WHO. Cuban international trade in health services is made possible by its competitive research in specific areas of medicine and medical informatics, the quality of its traditional and virtual education and health care services, its high concentration of physicians and health care professionals, its health care information and telecommunications technologies, and the exportability of certain aspects of its social medicine model to industrialized as well as developing countries.

First, in the *cross-border service delivery* mode, Cuba continues to develop its considerable potential for electronic delivery of information, telemedical services, and medical education primarily through INFOMED and specialized virtual infrastructures. The Cuban Virtual Library in collaboration with the Latin American and Caribbean Center on Health Sciences Information of the Pan American Health Organization and the Brazilian Virtual Library delivers an important information resource, much of which is freely accessible on the Internet. The Cuban collection of specialized medical journals presents the results of Cuban research and accounts of the Cuban health care system experience. Foreign authors are also invited to contribute articles to be published in Spanish, thus creating a controlled medical information market. The Virtual University offers Cuban [82] and international content through the Internet as well as a forum through the Virtual Clinic for expert consultation with physicians and health care professionals associated with the University [83].

Because of trade restrictions under the US embargo and other resource constraints, electronic trade in information and education is more highly developed than conventional cross-border trade. However, some publications as well as biotechnology and pharmaceutical products may be purchased as advertised online and delivered by regular mail services. Examples are products offered through Cuban research institutes [84] and represented on external websites such as the International Center for Scientific Research [85], a free public utility service based in France.

The second mode of health services trade, *consumption abroad,* is a very important component of Cuban international trade. Consumers from both industrialized and developing countries go to Cuba to receive health care services as well as training and education in disciplines related to medicine. High-quality health care is available at competitive prices, particularly innovative treatments for conditions for which care is unavailable in other countries, such as pigmentary retinopathy or vitiligo [86]. Again, Cuban research in medicine is the foundation of this international competitive advantage in offering certain very specialized care. Medical care is offered freely or under public subsidy to patients from certain countries with which Cuba maintains bilateral agreements on social security, while luxury services such as cosmetology are offered at US dollar rates, as well as combined health care and tourism packages for foreign clientele [87]. This trade in health care services is led by Cubanacán [88], the Cuban holding company dedicated to tourism, through SERVIMED, a specialized trading company founded in 1994. Sales of services to foreigners yielded revenues of US $20 million dollars in 1996, increasing to US $30 million in 1998 [86]. MINSAP projections estimate potential sales of such services at US $60 million [89,90]. These services are advertised through the INFOMED Portal.

Also, in the consumption abroad mode, students receive training and education in medicine and related disciplines at Cuban educational institutions and specialized clinics presented online. While some students receive subsidized education, generally fees are paid in US dollars at very competitive rates, thus attracting students from all over the world and generating significant foreign exchange earnings [91]. Again, it is important to note that these students come from industrialized nations as well as developing countries. The Latin American School of Medical Science (Escuela Latinoamericana de Ciencias Médicas) [92] was created in 1998 to respond to the regional shortage of trained physicians made apparent by hurricane Mitch. This school has trained as many as 6000 medical students from the Americas and Africa and even promotes applications from US citizens through SeattleCuba.org, an organization for friendship and cultural exchange between the people of Seattle, Washington and Cuba [93]. Scholarships are offered to students who agree to serve poor communities upon return to their own countries. Recently, scholarships for 500 students from the United States were set up with the objective of contributing to representation of ethnic and other communities that are underrepresented in the medical professions [94,95].

A final type of service trade in consumption abroad is the organization of international workshops, seminars, and conventions for both scholarly, educational, and commercial (eg, trade fairs) objectives related to health care. These events are advertised through INFOMED and attract participants from around the world, thus bringing significant foreign exchange revenues [96]. Research in health care is also a part of the Cuban Science and Technology Forum organized throughout the country to promote innovative solutions to problems in applied science. Although this event has in the past been restricted to Cuban institutions, recent efforts have focused on internationalizing the competition and publishing its results [97].

One of the objectives of the first international extension of the Science and Technology Forum, the Symposium on the Impact of Science and Technology on Cuban Health (El Simposium: Impacto de la Ciencia y la Innovación Tecnológica en la Salud Cubana) in 2000, was to attract joint international projects and foreign investment in the Cuban national health care system, thus encouraging the third mode of trade in health care services through some controlled foreign *commercial presence.* Foreign investment relevant to the health care system has contributed to its telecommunications infrastructures as well as availability of international information resources such as Medline.

Although the amount of investment in such activities cannot be reliably estimated, growing joint enterprises with foreign firms in medical research and biotechnology contribute significantly to research, development, and international marketing of new Cuban products [98,99]. Development of Cuban biotechnology is led by the Cuban Center for Genetic Engineering and Biotechnology (CIGB) [100] and the Western Havana Bio-Cluster of 52 specialized institutions [101]. The Cuban strategy in research and development in the field of biotechnology incorporates both physically proximate institutional clusters and virtual infrastructures [25,26,102]. The Western Havana Bio-Cluster offers a physical environment fostering inter-organizational exchange of research and ideas, while the CIGB promotes development of an extended network of collaborators through an Internet presence.

The CIGB Business Development Group negotiation policy for alliance agreements outlines conditions regarding scientific collaboration and business investment in research and development of new pharmaceuticals [103]. The CIGB has made its Isotopica software freely available to registered users as a Web application for research in the field of proteomics (the study of the structure and functions of proteins) with the collaboration of the Japanese Institute for Protein Research of Osaka University [104]. This offering extends opportunities for research collaboration with partners in the developed as well as the developing world. Other institutions contributing to Cuban trade in biotechnology include the Finlay Institute [105] and the National Center for Scientific Research [106].

The fourth mode of trade, *movement of health personnel,* is also critical to Cuban foreign policy as well as international health care services trade. Cuba’s high concentration of well-trained, relatively low-cost physicians and other health care professionals makes possible their mobilization in a strategy of assistance to developing countries experiencing shortages of such personnel. Cuba is one of several countries, including India, the Philippines, and Egypt, where education and training of health care personnel exceed country requirements. In the case of Cuba, these personnel contribute to disaster relief efforts and other services in developing countries [107]. While these activities for development assistance may bring limited revenues, they extend Cuban influence and leadership in the developing world. Cuban schools and clinics have also been opened to serve students and patients in some Latin American and African countries. For example, SERVIMED opened a Cuban hospital in Brazil with the participation of Brazilian investors to respond to local demand for treatment of skin disorders [108,109]. In the future, MINSAP will focus greater efforts to provide remunerated advisory and consultancy services in medicine, medical informatics, and health care system design and management [86].

## Discussion

Analysis of Cuban international trade in health care services shows the importance of its telecommunications infrastructures and expertise in medical informatics for service promotion and delivery. Despite many political and economic challenges, Cuba has developed a significant presence in international health care services markets and collaborative activities with the developing countries of the Caribbean and Latin America. Of particular importance is the coherence between design of the system and the socialist ideological values of its institutions: the ethic of universal and free access to health care services as well as attention to the collective social and environmental dimensions of health [110]. The unique features of the Cuban model enhanced by INFOMED and virtual infrastructures include the following:

- A systems perspective integrating health care service delivery, research, information resources, and education
- Horizontal coordination and integration through INFOMED and telecommunications infrastructures with vertical control through MINSAP and government hierarchy
- Government and health care administration serving socialist ideology: social control and universal citizen access
- Emphasis on individual assessment and community health evaluation including physical and mental health dimensions
- Priority on holistic, preventive health care in the family context rather than in specialized health care institutions
- Emphasis on original research and innovation in medicine, medical informatics, health care management, and related disciplines
- Recognition of the importance of methodological considerations in elaboration of data collection and information systems
- Effective mobilization of information and telecommunications technologies to achieve horizontal and interdisciplinary integration of the health care system and to promote Cuban contributions to international health care services trade
- Dual health care service market structure with emphasis on open information markets in education, research, and practice supporting trade on international services markets through the Internet
- Through the Virtual University, strong emphasis on training and continuing education of highly qualified physicians and other health care personnel as well as their indoctrination with values supporting service for the collective good

The Cuban approach to health care could be characterized as “high tech-high touch,” integrating the family and community context in individual assessment and risk evaluation. Both the high concentration of health care professionals and the highly developed telecommunications and information systems of INFOMED contribute to this strategy. In the Cuban ideology, health care is viewed as a social process and a responsibility distributed throughout all levels of society [111,112]. This model suggests some important questions with respect to the globalization of health care services markets. Cuba has developed a significant presence in that global market, but one of the risks is emergence of a dual standard of service differentiating health care reserved for Cuban citizens from services offered to patients remitting foreign currencies on international markets. This risk is associated with the difficulties of integrating systems based on diverse ideologies—socialism and capitalism—on a global level.

The transferability of the Cuban model to other national settings is contingent upon interpretation of values such as individual privacy and intellectual property [113]. Regulation of world trade in health care services has favored privatization of the sector, and the future of ideological diversity in the global economy is a topic of intense debate [80]. More extensive qualitative case analyses of complex health care systems will contribute to better understanding of ideological diversity and the role of telecommunications and virtual infrastructures in the integration of global health care markets.

## Abbreviations

AIDS: acquired immunodeficiency syndrome
BIREME: Brazil-based Latin American and Caribbean Center for Information Sciences
BVS: Biblioteca Virtual en Salud
CIGB: Cuban Center for Genetic Engineering and Biotechnology
CNICM: National Information Center for the Medical Sciences (Centro Nacional de Información de Ciencias Médicas de la República de Cuba)
GNP: gross national products
MINSAP: Cuban Ministry of Public Health (Ministerio de Salud Pública)
SCIELO: Latin American Scientific Electronic Library Online
WHO: World Health Organization
